# The Heart Failure Optimization Study (HF-OPT): rationale and design

**DOI:** 10.1007/s00399-022-00920-5

**Published:** 2023-01-25

**Authors:** R. Sanchez, D. Duncker, B. Colley, M. Doering, S. Gummadi, C. Perings, M. Robertson, G. Shroff, C. Veltmann, Heart Failure Optimization Study (HF-OPT) Investigators

**Affiliations:** 1 HCA Florida Heart Institute, St. Petersburg, FL USA; 2 Hannover Heart Rhythm Center, Hannover Medical School, Hannover, Germany; 3 Jackson Heart Clinic, Jackson, MS USA; 4 Heart Center Leipzig, University of Leipzig, Leipzig, Germany; 5 CVI of Central Florida, Ocala, FL USA; 6 Katholisches Klinikum Luenen, Luenen, Germany; 7 Trinity Medical, WNY, Buffalo, NY USA; 8 Baptist Heart Specialists, Jacksonville, FL USA; 9 Center for Electrophysiology Bremen, Klinikum Links der Weser, Senator-Wessling-Str. 1, 28277 Bremen, Germany

**Keywords:** Implantable cardioverter-defibrillator, Wearable cardioverter-defibrillator, Guideline-directed medical therapy, Left ventricular ejection fraction, Implantierbarer Kardioverter-Defibrillator, Tragbarer Kardioverter-Defibrillator, Leitliniengerechte medikamentöse Herzinsuffizienztherapie, Linksventrikuläre Ejektionsfraktion

## Abstract

**Background:**

According to the current guidelines, implantable cardioverter-defibrillators (ICD) for primary prevention in patients with heart failure and reduced ejection fraction (HFrEF) should not be considered until optimal guideline-directed medical therapy (GDMT) has been achieved for a minimum of 3 months. Optimization of GDMT often needs time beyond 3 months after diagnosis. The aim of the Heart Failure Optimization Study (HF-OPT) is to evaluate the recovery of left ventricular function beyond 3 months after diagnosis of newly diagnosed HFrEF.

**Methods:**

The HF-OPT multicenter study is comprised of two non-randomized phases (registry and study). During the first 90 days a wearable cardioverter-defibrillator (WCD) is prescribed and patients are enrolled in an observational pre-study registry. Registry subjects meeting inclusion criteria for the study portion at day 90 have ongoing left ventricular ejection fraction (LVEF) reassessment at 90, 180 and 360 days after the index hospital discharge, regardless of continued WCD use. Approximately 600 subjects will be enrolled in the study portion. Of those, one-third are anticipated to start the study phase at day 90 with reduced LVEF. The primary objective of this study is to observe the rate of recovery of LVEF > 35% between 90 and 180 days, while key secondary endpoints include mortality and WCD recorded arrhythmias and shocks.

**Discussion:**

The HF-OPT study will provide important information on the rate of additional recovery of LVEF > 35%, between 90 and 180 days, in newly diagnosed HF with reduced LVEF patients being titrated with GDMT. The results of the study may impact indications for primary prophylactic ICD implantation.

## Abstract (de)

**Hintergrund:**

Den aktuellen Leitlinien nach ist die primärprophylaktische Implantation eines implantierbaren Kardioverter-Defibrillators (ICD) bei Patienten mit Herzinsuffizienz und reduzierter linksventrikulärer Ejektionsfraktion (HFrEF) erst indiziert, wenn für mindestens 3 Monate eine optimierte leitliniengerechte medikamentöse Herzinsuffizienztherapie (LGMT) durchgeführt wurde. Die Optimierung der LGMT nach Erstdiagnose dauert allerdings häufig länger als 3 Monate. Ziel der Heart Failure Optimization Study (HF-OPT) ist es, die Erholung der linksventrikulären Funktion im Zeitraum jenseits von 3 Monaten nach Erstdiagnose einer HFrEF zu evaluieren.

**Methoden:**

Die multizentrische HF-OPT-Studie besteht aus zwei nichtrandomisierten Phasen: einer Register- und einer sich daran anschließenden Studienphase. In den ersten 90 Tagen wird den Patienten ein tragbarer Kardioverter-Defibrillator („wearable cardioverter-defibrillator“ [WCD]) verschrieben und sie werden in ein Beobachtungsregister aufgenommen. Bei Erfüllung der Einschlusskriterien für die Studienphase an Tag 90 erfolgen weitere Bestimmungen der linksventrikulären Ejektionsfraktion (LVEF) 90, 180 und 360 Tage nach erstmaliger Klinikentlassung, unabhängig von einer weiteren WCD-Verordnung. Ziel ist der Einschluss von etwa 600 Patienten in die Studienphase. Es wird erwartet, dass davon ein Drittel bei Tag 90 mit reduzierter LVEF in die Studienphase eintritt. Primärer Endpunkt der Studie ist die Erholungsrate mit LVEF > 35 % zwischen Tag 90 und 180. Zu den wichtigsten sekundären Endpunkten zählen die Mortalität sowie die durch den WCD dokumentierten Rhythmusstörungen und Schockabgaben.

**Diskussion:**

Die HF-OPT-Studie wird wertvolle Daten zur Erholung der LVEF auf > 35 % zwischen Tag 90 und 180 bei Patienten liefern nach Erstdiagnose einer HFrEF unter einer LGMT. Die Ergebnisse können die Indikationsstellung zur primärprophylaktischen ICD-Implantation beeinflussen.

## Introduction

Implantable cardioverter-defibrillators (ICDs) have been shown to reduce both arrhythmic and total mortality in stable heart failure (HF) patients [1] with reduced ejection fraction (EF). However, these implantable devices contain a certain level of risk, and according to the ACC/AHA/ESC guidelines ICDs for primary prevention HF patients should not be considered for implantation until optimal guideline-directed medical therapy (GDMT) has been achieved for a minimum of 3 months [2, 3]. As medication titration is challenging to achieve GDMT levels within 90 days after discharge, ICD implantation during this period does not meet the current GDMT standards.

Besides ICDs and medical therapy, another option of sudden cardiac death (SCD) protection frequently used after HF hospitalization is the wearable cardioverter defibrillator (WCD) [4]. WCDs have been commercially approved for use by the Food and Drug Administration (FDA) and have been CE-marked for Europe since 2001. Since a WCD is non-invasive it makes an ideal device for protecting HF patients in the case of a temporary high SCD risk, but recovery is still possible. After medications have been titrated to appropriate target doses and the EF has been given time to improve, the WCD can be removed and an assessment for ICD implantation due to ongoing SCD risk can be performed. Titration to therapeutic levels is generally not achieved within three months of initiation [5, 6] for some drugs known to improve ventricular dysfunction, and improvements in heart failure symptoms and ventricular function may occur after optimization of medical therapy over time. Recent findings of the PROLONG registry show that 33% of patients with non-improved low LVEF at 3 months may indeed improve LVEF to ≥ 35% by extending heart failure optimization therapy beyond the 3‑month waiting period [7].

The primary objective of this study is to observe the rate of recovery of left ventricular function (defined as EF > 35%) between this later time period of 90 and 180 days in newly diagnosed HF with reduced EF patients who were prescribed the WCD while being initiated on HF medications. This will be measured from LVEF at hospital discharge, and then regularly at 90, 180 and 360 days after WCD start, regardless of continued WCD use, prior LVEF improvement, or ICD implantation. While approximately one-third of the patients will likely experience LVEF recovery in the first 90 days [7], we hypothesize that an additional 5–10% of patients will experience EF improvement between 90 and 180 days as GDMT is achieved. This would be considered clinically significant in terms of the potentially avoided primary preventive ICD implantations.

## Methods

### Study design

The HF-OPT study is a multi-center prospective observational study of newly diagnosed HF with reduced EF patients to test the hypothesis that additional LVEF recovery occurs between 90 and 180 days as GDMT is achieved (ClinicalTrials.gov Identifier: NCT03016754). This study is being conducted according to US and international standards of good clinical practice and has been approved by all relevant medical ethics committees. A total of 67 centers with WCD prescribing physicians were selected for the study based upon expressed interest. All sites were in the US or Europe and included both academic and community centers.

**Fig. 1:**
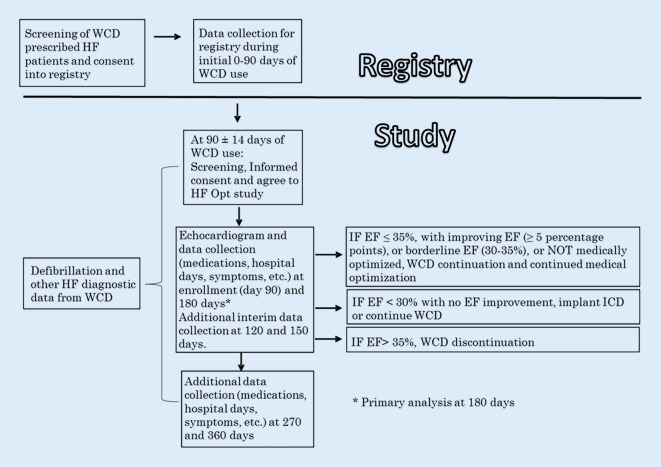
Study flow diagram

Although the study phase does not start until day 90, all eligible, consenting patients will be entered into a registry at the start of WCD use. The pre-study registry will allow the collection of early (90 day) outcomes and data in those patients that are likely to be eligible for the study at day 90, or that are eligible but refuse the study at day 90. While the primary endpoint analysis is at day 180, subjects will be followed for 360 days. See Fig. 1 for study flow diagram. The study is sponsored by the WCD manufacturer (ZOLL, Pittsburgh, PA, USA).

### Objectives

#### Primary endpoint

Descriptive or summary statistics of serial LVEF measurements will be used to analyze the rate of recovery of ventricular dysfunction following discharge. LVEF at 180 days will be compared to the means of LVEF at baseline and 90 days. The percentage of patients reaching the goal of LVEF > 35% will be compared at 90 days and 180 days. Also, the time taken to reach LVEF > 35% will be calculated for those who improve and compared between groups (based on baseline characteristics). The core lab determinations will be used to verify local estimates of LVEF and may be used in secondary analyses of the study data.

Descriptive statistics will be used to assess the degree GDMT (% of target dose) was reached at 90 and 180 days, and how GDMT relates to LVEF recovery. We will also assess how self-reported adherence to the GDMT regimen affects LVEF recovery and overall clinical improvement.

#### Secondary endpoint: treatments by the WCD or ICD

Effectiveness of the WCD in treating ventricular arrhythmias will be evaluated through descriptive means. Clinical outcomes and device data from any defibrillation or cardiac event will be reviewed for success as defined by appropriate detection and defibrillation, conversion out of the detected ventricular arrhythmia, and consciousness when medically evaluated after treatment.

For the safety endpoint, descriptive statistics will be used to analyze the incidence of unnecessary shocks from WCD and ICD in this population. Causes of prolonged false detections and lack of response button use in WCD users will be reviewed.

#### Secondary endpoint: incidence of all untreated sustained VT/VF events during WCD use not associated with a shock event

Descriptive statistics will be used to analyze the incidence of untreated sustained (≥ 30 s) episodes of VT or VF. All possible reasons for a lack of shock will be reviewed, including response button use, rate threshold settings, or ECG artifact. ECG recordings will be adjudicated by a committee independent of the sponsor.

#### Secondary endpoint: incidence of all sudden cardiac arrest (SCA) events

Descriptive statistics will be used to analyze the incidence and etiology of SCA from the beginning of WCD use to end of study. “Sudden Cardiac Death” will be defined as an unexpected, non-traumatic, non-self-inflicted fatality in otherwise stable subjects who die within 1 h of the onset of the terminal symptoms. Subjects dying more than 1 h after a sudden cardiac arrest (SCA) from a ventricular arrhythmia will be designed as non-sudden death due to ventricular arrhythmia. For unwitnessed deaths participants will meet the definition of sudden cardiac death if they are found dead within 24 h of being well, assuming there is no evidence of another cause of death during that time period. Autopsy results may be used when available.

#### Secondary endpoint: incidence and etiology of other WCD recorded arrhythmias, such as asystole and sustained (≥ 30 s) supra-ventricular arrhythmias

Descriptive statistics will be used to analyze the incidence of non-VT/VF events that are recorded by the WCD. Such arrhythmic events will be evaluated for correlation with the other endpoints. ECG recordings will be adjudicated by a committee independent of the sponsor.

#### Secondary endpoint: mortality

Descriptive statistics will be used to analyze the effect of WCDs on 180-day and 360-day mortality following discharge from HF. Kaplan-Meier curves and survival analysis will be performed both for aggregate mortality and independently for sudden and non-sudden cardiac deaths. Cause of mortality will be adjudicated by a committee independent of the sponsor. The influence of baseline characteristics and EF recovery status on mortality will be evaluated. Comparisons will be made using the Seattle Heart Failure model as a predictive tool of post-discharge mortality.

#### Safety endpoint: complication data from WCD or ICD

Descriptive statistics will be used to analyze the incidence of complications from WCDs and ICDs in this population. This will include inappropriate treatments, as well as any other complications that may lead to hospitalizations or death.

### Population and recruitment

#### Eligibility and recruitment.

Patients were enrolled from March 2017 through January 2021. Prior to 90 days, eligible patients with the WCD are enrolled into a registry. Registry patients are enrolled during the first 30 days of WCD use. The population for this study group are subjects who had an LVEF ≤ 35% following hospitalization for newly diagnosed HF, and who have already been prescribed a WCD for 90 days. Main inclusion and exclusion criteria are listed in Table 1. The study target is to enroll approximately 1400 subjects in the registry portion in order to achieve 600 subjects for the study period at 90 days. Subjects will be enrolled from study sites in the US, Germany, Austria, and France.

**Table 1:** Main inclusion and exclusion criteria

**HF-OPT: Registry 0–90 Days**
*Inclusion Criteria*
Newly diagnosed heart failure patients with a first-time hospitalization for HFrEF (≤ 30 days since a first HF hospitalization) prescribed the WCD
Ischemic or non-ischemic cardiomyopathy
EF ≤ 35%
Subjects ≥ 18 years of age
*Exclusion Criteria*
Active unipolar pacemaker
Physical or mental condition that could impair subject’s ability to properly interact with the device
Currently participating in an interventional clinical study
Any skin condition that would prevent subject wearing the WCD
Advanced directive prohibiting resuscitation
**HF-OPT: Study 90–360 Days**
*Inclusion Criteria*
Subjects that participated in the 90-day HF-Optimization Registry
Subjects that have used the WCD for 90 ± 14 Days
OR
Subjects enrolled in the Registry after March 1st, 2019 and received an ICD prior to 90 days of WCD use
*Exclusion Criteria*
All of the same Exclusion Criteria as Registry
AND
Subjects that have a QRS duration of ≥ 135 ms. and are planned for cardiac resynchronization therapy during the study duration
Subjects with recent myocardial infarction or coronary revascularization (since start of WCD wear; i.e., 0–90 days of WCD wear)

*HFrEF* Heart failure with reduced ejection fraction, *EF* ejection fraction, *WCD* wearable cardioverter-defibrillator, *HF* Heart failure, *ICD* implantable cardioverter-defibrillator

### Study procedures and follow-up

The study procedures timeline is outlined in Table 2.

**Table 2:** Evaluation schedule

**A: Study Procedures Table for WCD Pre-study Registry**							
				*Timing of data collection (post WCD start)*			
*Data collected*				*Initial visit (0–30* *days)*		*WCD end*	
Screening				X		–	
Baseline demographic data				X		–	
Echo data				X		–	
Medications				X		X	
WCD use and discontinuation data (medical or patient reason for ending use, compliance)				–		X	
Defibrillation data: WCD (Appropriateness, consciousness, effectiveness, clinical status)				–		X	
**B: Study Procedures Table for HF-OPT Study**							
*Data collected*	*Enrollment* *visit (90* *days)*	*120* *Days* *call*	*150* *Days* *call*	*180* *Days* *visit*	*270* *Days* *visit*	*360* *Days* *visit*	*WCD end*
Screening	X	–	–	–	–	–	–
ECG	X	–	–	–	–	–	–
Follow-up echo data	X	–	–	X	–	X	X^a^
Clinical status (medical care utilization, medications, symptoms, ICD implantation, SCA)	X	X	X	X	X	X	X
WCD use and discontinuation data	–	–	–	–	–	–	X
Defibrillation data: ICD or WCD (Appropriateness, consciousness, effectiveness, clinical status)	X	X	X	X	X	X	X
HF diagnostics (activity, position, heart rate metrics)	–	–	–	–	–	–	X
Long-term survival status	–	–	–	–	–	X	X

^a^If echo performed at end of WCD use, and not at one of the pre-specified timepoints

#### Pre-study registry

Patient demographics, limited medical history, medications, laboratory results, imaging, invasive and non-invasive cardiac examinations were collected upon registry entry. WCD device records were interrogated to determine compliance with use, defibrillation events, and arrhythmia detection. Reasons for terminating WCD use were also collected (Table 2).

#### Study period

Follow-ups were made in person or via phone at days 90, 120, 150, 180, 270 and 360 post WCD start.

### Data collection

Data will be collected at the investigational sites using the electronic data capture (EDC) system ClindexLIVE (Fortress Medical Systems, LLC, Hopkins, MN, USA). Data will be entered at the investigational sites by trained staff. Entered data will be reviewed by the site investigator, who will affirm its accuracy and completeness by electronic sign-off. The sponsor will manage and monitor the data for quality.

### Statistical analysis

#### Sample size estimation

**Fig. 2:**
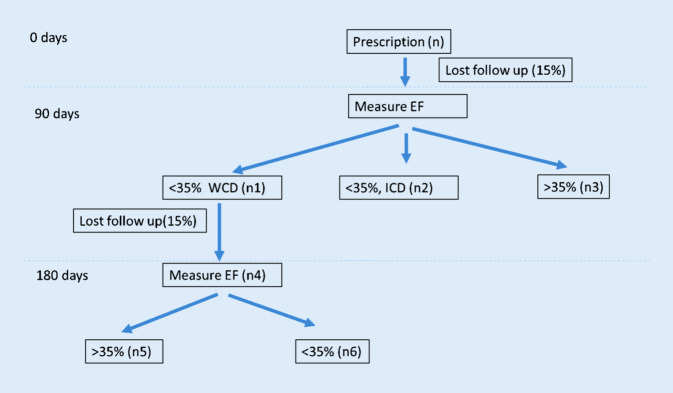
Flow chart for sample size estimation

This dataset will be used to evaluate among all patients whether more than 5% of them will experience EF improvement between 90 and 180 days (n_5_ / *n* > 5%) (Fig. 2).

To calculate the number of samples (*n*), the authors first calculated *n*_4_, then calculated *n* based on the following formula:

$$n=\frac{n_{4}}{P_{\,\text{followup}2}}*\frac{1}{p_{\mathrm{wcd}}}*\frac{1}{P_{\,\text{followup}1}}$$

where *p*_*wcd*_ is the percentage of patients that will wear WCD beyond 90 days (n_1_ / (n_1_ + n_2_ + n_3_)), and *p*_*followup*1_ and *p*_*followup*2_ are the percentage of patients with proper follow up from 0–90 days and from 90–180 days, respectively.

To calculate sample size for n_4_, the authors performed a superiority test. The statistical hypothesis to be tested is:

$$H0\colon P\leq P_{0}+\delta$$

vs:

$$H1\colon P> P_{0}+\delta$$

The sample size of the superiority test is calculated as the following formula:

$$n_{4}=p\left(1-p\right)\left(\frac{Z_{1-\alpha }+Z_{1-\beta }}{p-p_{0}-\delta }\right)^{2}$$

where *p* is the actual proportion of patients that will have EF improvement (n_5_ / n_4_). *p*_0_ is the baseline proportion and specified as 0.001 (close to 0). δ is the test margin, since the hypothesis is that more than 5% of them will experience EF improvement between 90 and 180 days (n_5_ / *n* > 5%), δ depends on *p*_*wcd*_ as $\delta =0.05/P_{\mathrm{wcd}}$. The authors also specify α as 0.05, β as 0.1.

As shown in Fig. 2, sample size (*n*) depends both on *p*, the actual proportion of patients that will have EF improvement (*p* = n_5_ / n_4_) and *p*_*wcd*_, the percentage of patients that will wear WCD beyond 90 days ($p_{\mathrm{wcd}}=$ n_1_ / (n_1_ + n_2_ + n_3_)). In this study, the authors specify α as 0.05, β as 0.1. Based on their assumptions regarding patients that will have continued WCD wear from 90–180 days, and the proportion of WCD patients at 90 days to have further EF improvement, the authors plan to prescribe WCD to 1400 patients (initial registry sample size), in order to have approximately 600 patients eligible for the study at day 90.

## Discussion

Previous clinical data reveals that the following questions remain controversial subjects in newly diagnosed HF patients:

1. How often is optimal medical therapy achieved in a real-world setting?
2. When to implant an ICD in a newly diagnosed patient?
3. How often and when to measure LVEF recovery?

Titration to therapeutic levels is generally not achieved within 3 months of initiation. For some drugs known to improve ventricular dysfunction, improvements in heart failure symptoms and ventricular dysfunction may occur after optimization of medical therapy over time [8].

A predecessor of the HF-OPT Study was the Prolongation of Reverse remOdelling period to avoid untimely ICD impLantation in newly diagnOsed heart failure usiNG the wearable cardioverter defibrillator (PROLONG) study. The aim of the retrospective PROLONG study was to analyze evolution of LVEF after first diagnosis of a reduced LVEF ≤ 35% during elaborate optimization of heart failure therapy using the WCD to provide a secured prolongation of the observation period. They found 33% of patients that still had low LVEF at 3 month follow-up had improved during the prolongation period beyond 3 months after diagnosis, emphasizing the need for elaborate optimization of heart failure therapy [7]. Importantly, none of the patients not receiving an ICD experienced SCD or malignant arrhythmias during an extended follow-up of several years [9]. The authors sought to determine whether these results could be confirmed in a large, multi-center, prospective study, with set time-points for LVEF assessment and follow-up.

One potential reason for a slow or inadequate recovery of LVEF may be a lack of reaching target doses of GDMT during the first 3 months [10–12]. A retrospective study of Medicare patients linked to the National Cardiovascular Data ICD Registry showed a very low rate of GDMT being achieved in the 90 days before ICD implantation [13]. Only 61.1% had filled their prescriptions for an HF beta-blocker plus an angiotensin-converting enzyme inhibitor or angiotensin receptor blocker at least once during the 90 days before ICD implantation, and only 28.3% had a supply for ≥ 80% of the 90 days. Interestingly, those with the shortest duration of HF and most recent evaluation of LVEF were the least likely to receive GDMT. Death within 1 year occurred more frequently in those patients not receiving any GDMT even after adjusting for patient characteristics, HF severity, and comorbidities. More recent registries, such as the CHAMP-HF and CHECK-HF, have confirmed that GDMT is still being prescribed at lower than recommended doses [5, 6]. This may become even more of a problem as newer HF medications are approved and added on top of this list of GDMT medications. Also, just because the proper dosage may be prescribed does not mean the patient is taking it as directed.

Although LVEF is typically measured in the beginning, and usually at some follow-up period, it is unknown how often LVEF should be measured and what “recovery” of LV dysfunction means in terms of outcomes. To support our primary hypothesis that an additional 5% of patients will experience LVEF improvement between 90–180 days, LVEF measurement for all patients at 90 (±14), 180 (±14) and 360 (±14)-days after WCD start will be requested and recorded. For this study, LVEF > 35% will be considered as LVEF improved. This study is unique in that the LVEF trajectory, GDMT as a percent of target dose, and outcomes will be tracked for all subjects, regardless of ICD implantation. This will allow us to observe how the GDMT regimen, and self-reported adherence, relates to longitudinal LVEF recovery and clinical outcomes.

## Conclusion

The HF-OPT study will provide important information on the rate of additional recovery of LVEF > 35%, especially between 90 and 180 days, in newly diagnosed HF with HFrEF patients being treated with GDMT, and will hopefully translate to better management of GDMT in newly diagnosed HF patients.

## Appendix

### Trial organization

- ZOLL Cardiac Management Solutions, Pittsburgh, PA

### Participating countries

- Austria,
- France,
- Germany,
- USA.

### Core laboratories

- Echo core lab Europe: Department of Cardiology, Hannover Medical School, Carl-Neuberg-Str. 1, 30625 Hannover, Germany.
- Echo core lab USA: Medpace, Inc., 5375 Medpace Way, Cincinnati, Ohio 45227 USA

## Declarations

All procedures performed in studies involving human participants or on human tissue were in accordance with the ethical standards of the institutional and/or national research committee and with the 1975 Helsinki declaration and its later amendments or comparable ethical standards. The study was approved by the ethics committee of Hannover Medical School (No.: 7292) and by the ethics committee by each participating center. Clinical trial registration: NCT03016754. All patients gave written informed consent to HF-OPT.
