# Publisher Erratum: The Heart Failure Optimization Study (HF‑OPT): rationale and design

**DOI:** 10.1007/s00399-023-00928-5

**Published:** 2023-02-14

**Authors:** R. Sanchez, D. Duncker, B. Colley, M. Doering, S. Gummadi, C. Perings, M. Robertson, G. Shroff, C. Veltmann, Heart Failure Optimization Study (HF-OPT) Investigators

**Affiliations:** 1 HCA Florida Heart Institute, St. Petersburg, FL USA; 2 Hannover Heart Rhythm Center, Hannover Medical School, Hannover, Germany; 3 Jackson Heart Clinic, Jackson, MS USA; 4 Heart Center Leipzig, University of Leipzig, Leipzig, Germany; 5 CVI of Central Florida, Ocala, FL USA; 6 Katholisches Klinikum Luenen, Luenen, Germany; 7 Trinity Medical, WNY, Buffalo, NY USA; 8 Baptist Heart Specialists, Jacksonville, FL USA; 9 Center for Electrophysiology Bremen, Klinikum Links der Weser, Senator-Wessling-Str. 1, 28277 Bremen, Germany

Erratum to:

Herzschr Elektrophys 2022

10.1007/s00399-022-00920-5

Please note that only C. Veltmann writes on behalf of Heart Failure Optimization Study (HF-OPT) Investigators. The original article has been corrected.

